# Challenges in the Endotracheal Intubation of a Patient With Severe Spine Curvature Abnormality

**DOI:** 10.7759/cureus.18161

**Published:** 2021-09-21

**Authors:** Hanya Javaid, Shiza Shafique, Komal Ajay, Fahad Zubair, Syed Farjad Sultan

**Affiliations:** 1 Anesthesiology and Critical Care, Civil Hospital Karachi, Karachi, PAK; 2 Internal Medicine, Civil Hospital Karachi, Karachi, PAK

**Keywords:** difficult intubation, trachea, difficult airway, kyphosis, scoliosis, lordosis, spine curvature disorders, anesthesiology, emergency medicine

## Abstract

Severe spine curvature disorders are commonly associated with multiple pathophysiological challenges during airway management, secondary to physiological and anatomical factors. These factors are mostly related to the reduced vital capacity and chest wall compliance, along with the misalignment of axes and limitation in neck movement. Careful assessment and planning of alternative strategies by experienced anesthesiologists, appropriate positioning, and proper use of rescue devices can significantly improve the chances of successful intubation. In this report, we present a case of a 26-year-old man with severe spine curvature abnormality, unstable vitals, low Glasgow Coma Scale (GCS) score, and low oxygen saturation necessitating emergency intubation. We shed light on the importance of proper airway assessment and good team communication and also highlight the technique used for emergency intubation in case of an anticipated difficult airway.

## Introduction

The human spine has a natural degree of the inward curve in the cervical and lumbar region ranging between 35 and 80 degrees and an outward curve in the thoracic region ranging between 30 and 50 degrees [1]. These curves help the spine withstand stress from movement and gravity. When the curvature is misaligned or exaggerated, it results in abnormal convex, concave, or lateral curves, namely, kyphosis, lordosis, and scoliosis [2]. Spine curvature disorder has a prevalence of 0.3% to 15.3%, with a female to male ratio of 3:1 [3]. The most common site for this disorder is the thoracolumbar spine. It can also affect the cervicothoracic region [1]. It results in pathophysiological changes that present various challenges for the anesthesiologist. The clinical impact of this disorder depends on factors like the degree of axial rotation, the number of vertebrae involved, and the severity and site of the abnormality in curvature. The involvement of the cervical vertebrae may result in difficult airway management and reduced range of neck movements. The difference in anatomical structure may be associated with increased morbidity secondary to prolonged and multiple attempts at laryngoscopy and tracheal intubation [3]. The involvement of the thoracic spine may cause rib cage deformity, which in severe cases leads to poor pulmonary function.

In the literature, few cases report the technique of intubation and anesthetic management in patients with kyphoscoliosis [4,5]. We present a case of a 26-year-old man with severe spine curvature abnormality, unstable vitals, low Glasgow Coma Scale (GCS) score (6/15), and low oxygen saturation, in need of emergency intubation.

## Case presentation

A 26-year-old man who was 50 kg in weight and 140 cm in height was scheduled for debridement of cellulitis, extending proximally from the sole of his right foot up to the middle third of his calf in the surgical unit. He developed sepsis and was consequently being treated with broad-spectrum antibiotics, which later changed to culture-sensitive antibiotics. The patient’s condition progressively deteriorated. Subsequently, on the 13th day of stay in the surgical unit, his vitals became unstable and his GCS score dropped. The department of anesthesia was called for the critical care and airway management of the patient.

**Figure 1:**
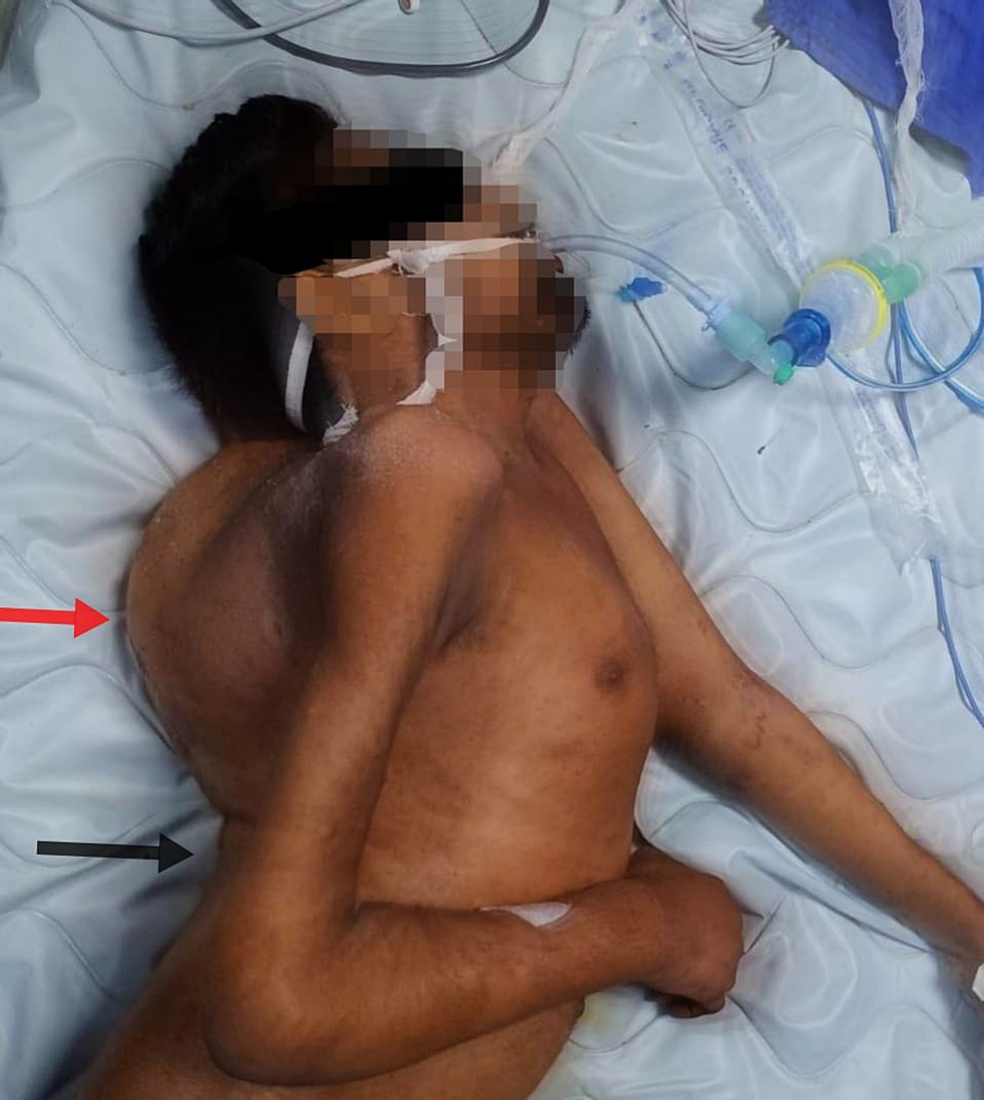
Patient lying in the left lateral position after endotracheal intubation, showing thoracic kyphoscoliosis (red arrow) and lumbar lordosis (black arrow).

**Figure 2:**
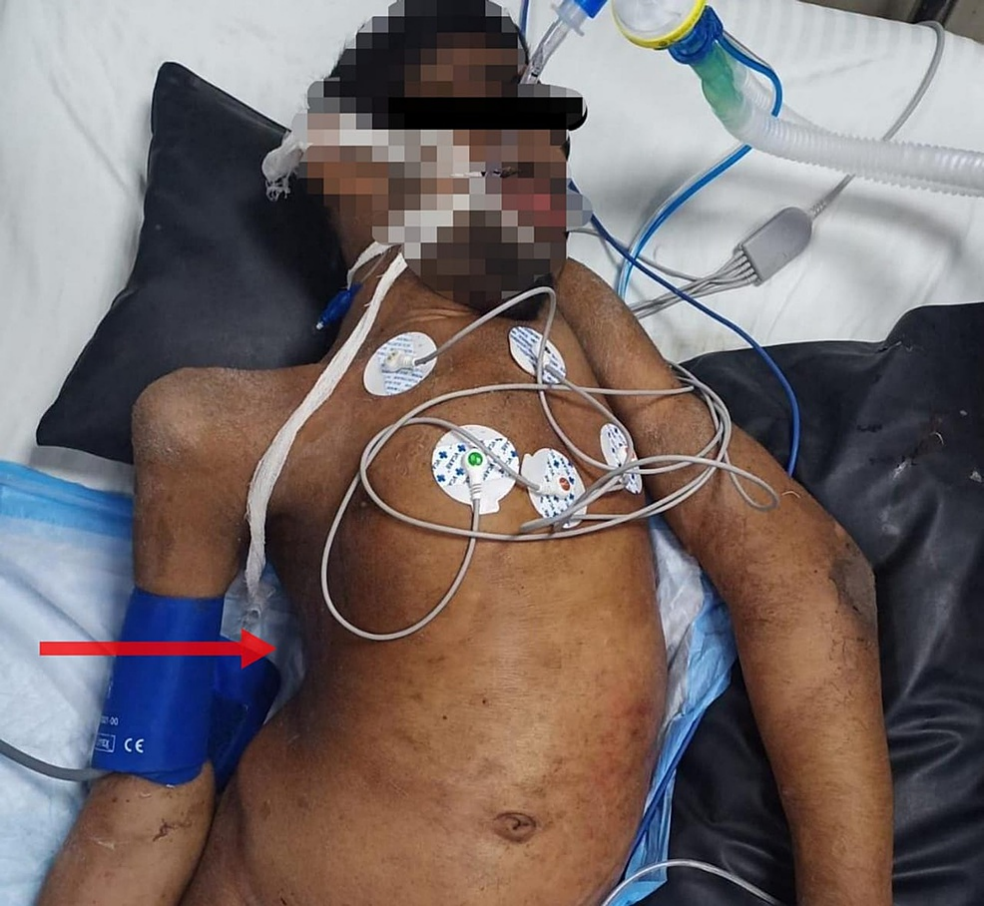
Patient lying in the supine position after endotracheal intubation, showing thoracic scoliosis (red arrow).

**Figure 3:**
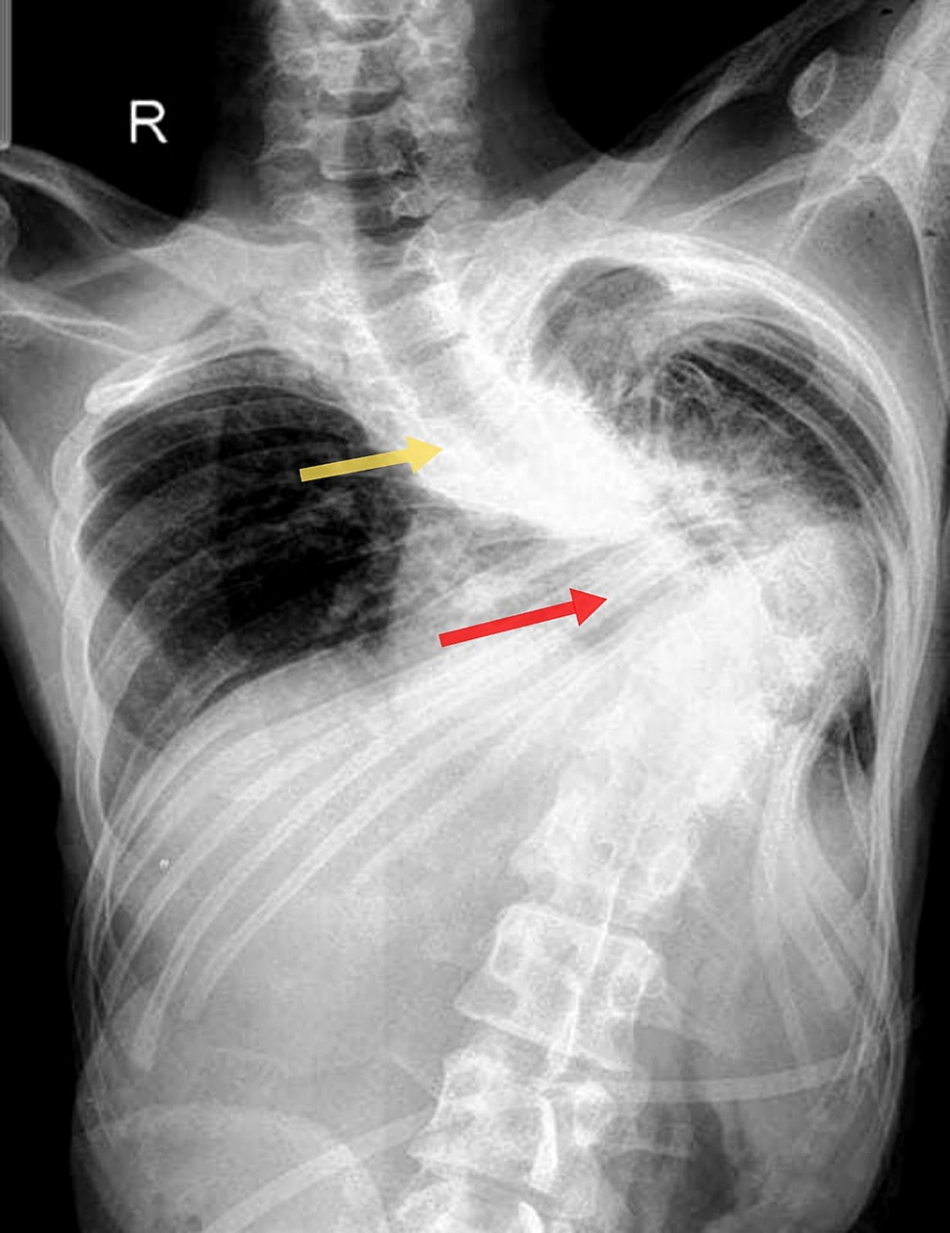
Chest X-ray shows tracheal deviation toward the left side of the chest (yellow arrow) and thoracic scoliosis (red arrow).

Upon assessment, the patient had a GCS score of 6/15 (E2V1M3), blood pressure of 80/40 mmHg, heart rate of 148 bpm, respiratory rate of 40 breaths/min, and oxygen saturation of 80% while on 15 liters of supplemental oxygen using a non-rebreather mask. On examination, his spine had curvature disorder with mild cervical kyphosis, severe thoracic kyphoscoliosis, and moderate lumbar lordosis resulting in the upper body contorting towards the left side and severe restriction of neck movements (Figures 1, 2). On auscultation, his cardiovascular examination was unremarkable. The chest examination revealed bilateral basal coarse crackles. The hematological and biochemical parameters were significant for leukocytosis, severe metabolic acidosis with a lactate of 5.8 mmol/L, and hypoxemia with a partial pressure of oxygen of 46 mmHg. Chest X-ray showed tracheal deviation toward the left side of the chest and a Cobb angle of 55° (Figure 3). The electrocardiogram (ECG) revealed a right axis deviation with T-wave inversion.

Emergency intubation was planned, along with resuscitation. The airway examination revealed a Mallampati classification of II and a restricted range of neck movement, especially during flexion. A team of anesthetists and assistants prepared the patient, attached the monitors, and checked the equipment for difficult intubation. Besides, the otorhinolaryngology (ENT) department was briefed about the case. Their team prepared for the "can't intubate can't ventilate" scenario and were in the standby position. The patient was propped with cushions below his left shoulder and the left lumbar region to optimize his position. He was given bag-mask ventilation initially but it was not effective as he started to desaturate. One of the anesthetists administered 2 mg of midazolam and 100 mg of suxamethonium before intubation. Subsequently, the senior anesthetist performed direct laryngoscopy using a size four blade and noted a Cormack-Lehane grade III view. A video laryngoscope was not employed due to a lack of availability. The tip of the epiglottis was marginally visualized and a gum elastic bougie (50 mm x 200 mm) was passed. Then an endotracheal tube of size 7.0 mm was railroad over the bougie. The chest was examined for the bilateral rise and auscultated for equal air entry in both lung fields. Due to unavailability, the capnometer was not utilized. Next, the endotracheal tube was fixed at 20 cm near the right oral commissure by an assistant. For management with ventilatory and vasopressor support, the patient was shifted to the intensive care unit.

## Discussion

Severe spine curvature disorders pose various challenges during airway management, secondary to physiological and anatomical factors.

Physiologically, the progressive nature of the disorder results in a combination of both obstructive and restrictive patterns of pulmonary disease due to limited vital capacity and chest wall compliance [4]. The abnormal curvature imposes an extrinsic restriction on the lung parenchyma and forces it into small spaces. That results in reduced vital capacity along with ventilation and perfusion ratio mismatch. Besides, the overall abnormality in the geometry of the thoracic cage produces a marked decrease in chest wall compliance that decreases the forced vital capacity and the forced expiratory volume in one second, consistent with an obstructive pattern [4]. The decline in functional residual capacity reduces the oxygen reserve. This leads to a shorter safe apnea period before the critical hypoxia develops. Second, the morphological features of the patient's anatomy lead to difficult intubation. The prerequisite for the translaryngeal insertion of an endotracheal tube includes optimal alignment of the oral, pharyngeal, and laryngeal axes, along with adequate visualization of pharyngeal and laryngeal structures during laryngoscopy [6]. In the case of severe deformity, there is a reduction in the articular mobility of the cervical spine that makes alignment of the axes difficult. Furthermore, during laryngoscopy, the laryngeal structures are hard to recognize and, the distortion of anatomy makes the successful placement of the endotracheal tube difficult.

In this case, we tried to minimize as many obstacles in emergency intubation as possible. First, a team of experienced and competent proceduralists was present, who anticipated the potential airway problems and discussed alternative strategies before any intervention. The otorhinolaryngology department was briefed about the case. Their team was near the surgical unit to take over in case of the "can’t intubate and can’t ventilate" scenario. Moreover, the anesthesiology team ensured that rescue devices like the oropharyngeal airway, nasopharyngeal airway, gum elastic bougie, stylet, and laryngeal mask airway were visible and immediately accessible. Several studies stress that improper planning, poor communication among team members, and unavailability of necessary equipment can make airway management and intubation difficult whereas the opposite is associated with better outcomes [6-8]. In this case, the patient was lying with the body contorted toward the left side, head displaced toward the side in which the spine was curved, asymmetrical shoulder and pelvic position, and round back. During intubation, the most common posture is of the neck flexed at approximately 35° and atlantooccipital joint extended at about 15°. It is known as the sniffing position and is as per the three-axis theory [9]. However, our patient had a restricted range of neck movement with slight extension and almost no flexion that made the alignment of axes hard. The positioning cushions were employed and strategically placed under the left shoulder girdle and the left lumbar region to minimize the degree of misalignment. This technique supports studies reported in the past that employed cushions for head positioning to visualize the glottis better [9,10].

According to Casey et al., the cardiopulmonary compromise in these patients results in hypoxemia and hypotension, which can further contribute to difficulty during intubation [8]. Therefore, crystalloids were administered to maintain pressure during the procedure and the patient was shifted later to the intensive care unit for vasopressor support. Lastly, successful endotracheal intubation in the first attempt is essential as it limits morbidity and the likelihood of adverse events secondary to repeated attempts. A gum elastic bougie was employed in the first attempt rather than after a possible failed attempt to minimize time loss and morbidity associated with repeated attempts. Driver et al. reported the higher first-attempt success rate of bougie compared to the use of endotracheal tube and stylet among patients undergoing emergency endotracheal intubation [11]. In this case, the small diameter of the bougie helped anticipate any unforeseen abnormal airway anatomy. In addition, a new generation of devices allows a better view of the glottis and successful placement of the endotracheal tube without direct visualization of the vocal cords. Devices such as video laryngoscopes allow endotracheal intubation without aligning the oral, pharyngeal, and laryngeal axes in patients with cervical spine abnormality [12]. However, these devices were unavailable in the resource-poor setting of our hospital and therefore could not be employed. The Difficult Airway Society guidelines (2015) were followed during the management of this patient [13].

## Conclusions

In conclusion, patients with severe spine curvature disorders present various challenges during anesthetic management that may be fatal in some cases. However, the risk of an adverse outcome or alternative invasive strategies can be curbed by employing various factors such as good communication, experienced team, accessibility of rescue devices, careful assessment, proper positioning, and utilization of appropriate airway devices to minimize time and morbidity associated with multiple attempts. Moreover, through new techniques and devices, such as the video laryngoscope, the view of the larynx is improved. This allows successful placement of the endotracheal tube without direct visualization of the vocal cords.

## References

[1] Issac S, Das JM (2021). Kyphoscoliosis. StatPearls [Internet].

[2] Kuo YL, Chung CH, Huang TW (2019). Association between spinal curvature disorders and injury: a nationwide population-based retrospective cohort study. BMJ Open.

[3] Saraçoğlu KT, Baygın Ö, Kafalı İH (2015). Kyphoscoliosis and difficult airway management. DBU Florence Nightingale J Med.

[4] Kundra P, Joseph A, Kumar S, Sai Chandran BV (2008). Double-lumen tube for ventilation in severe kyphoscoliosis. J Anesth.

[5] Kim HJ, Choi YS, Park SH, Jo JH (2016). Difficult endotracheal intubation secondary to tracheal deviation and stenosis in a patient with severe kyphoscoliosis: a case report. Korean J Anesthesiol.

[6] Janssens M, Hartstein G (2001). Management of difficult intubation. Eur J Anaesthesiol.

[7] George J, Kader JA, Arumugam S, Murphy A (2015). Successful intubation of a difficult airway due to a large obstructive vocal cord polyp augmented by the delivery of a transtracheal injection of local anaesthetic. BMJ Case Rep.

[8] Casey JD, Semler MW, High K, Self WH (2019). How I manage a difficult intubation. Crit Care.

[9] Hong HJ, Yun M, Kim SH, Hwang JW, Lee HC (2016). A pillow of 8 cm height did not improve laryngeal view and alignment of airway axes but increased anesthesiologist discomfort compared to a pillow of 4 cm height during tracheal intubation in adult patients. Korean J Anesthesiol.

[10] Dhar M, Karim HMR, Rajaram N, Prakash A, Sahoo SK, Narayan A (2018). A randomised comparative study on customised versus fixed sized pillow for tracheal intubation in the sniffing position by Macintosh laryngoscopy. Indian J Anaesth.

[11] Driver BE, Prekker ME, Klein LR (2018). Effect of use of a bougie vs endotracheal tube and stylet on first-attempt intubation success among patients with difficult airways undergoing emergency intubation: a randomized clinical trial. JAMA.

[12] Shirgoska B, Netkovski J (2012). New techniques and devices for difficult airway management. Acta Clin Croat.

[13] Frerk C, Mitchell VS, McNarry AF (2015). Difficult Airway Society 2015 guidelines for management of unanticipated difficult intubation in adults. Br J Anaesth.

